# Hospital and Institutional News

**Published:** 1911-12-02

**Authors:** 


### HOSPITAL AND INSTITUTIONAL NEWS.

### THE LIVERPOOL DISASTER.

The part played by the Northern Hospital, Liverpool, in the recent accident caused by the explosion at Messrs. Bibbys' works, was much misunderstood by the lay papers. These gave vivid accounts of "the nurses and staff rushing on the scene," but as Mr. K. W. Monsarrat, honorary surgeon to the hospital, pointed out, the hospital staff remained to cope with the work without appealing for assistance. A quarter of an hour after the accident 122 patients were received in the out-patients' hall. Most of them were terribly burnt, and five died there. No fewer than 76 were admitted to the wards, the remainder being treated and sent home with orders to attend as out-patients on the morrow. An hour and a half after the rush of cases every one had been attended to, and no patients were sent to other institutions. Dr. R. W. Murray gave a striking tribute to the behaviour of the wounded, for after attending to the injured, he wrote to the papers, saying that "so long as we have a fair sprinkling of the type of man treated at the Northern Hospital on (last) Friday afternoon, this country has not much to fear." The Northern Hospital's staffs, medical and nursing, deserve the warmest praise for the way in which they rose to the emergency.

### LONDON HOSPITALS AND NATIONAL INSURANCE.

The Central Hospital Council for London met at St. Thomas's Hospital last week, and after considering the effect upon the voluntary hospitals of the passing of the Insurance Bill as it stands at present, unanimously agreed: "1. That the effect upon the voluntary hospitals must be to diminish their income very seriously, and consequently to lessen the number of beds they will be able to maintain; as well as gravely to impair their efficiency as curative institutions, schools of medicine, and training schools for nurses. 2. That the Bill will be incomplete until it provides for the hospital treatment which insured persons must have if their medical needs are to be covered at all adequately by the Bill." The importance of this lies in its authoritative reiteration of the policy laid down by the British Hospitals Association. The attitude on the Insurance Bill of the voluntary hospitals is now laid down and agreed upon, so that they stand in one solid phalanx, pledged to the above demands and united in claiming them.

### THE BRITISH HOSPITALS ASSOCIATION.

The meeting of representatives of the voluntary hospitals of the United Kingdom, which will be held on Friday afternoon, 1st instant, after we go to press, should be memorable in the history of hospitals. The list of hospitals which will be represented covers every type, and includes workers all over the country. We hope the meeting will be conducted wisely and that those present will be content to vote rather than to speak. The most effective way to protect the interests of the voluntary hospitals would be for those present to combine in support of pressing the Government to make adequate provision for institutional—that is, hospital—treatment for insured persons. We have reason to hope the position has greatly improved within the last few days, for public interest is growing on this question, and should institutional benefit not be granted by the Government, any such inaction will probably lead to a social revolution of the most momentous importance. Success seems sure if the meeting is businesslike, the speakers are few and representative, and the whole proceedings unanimous and enthusiastic. Details cannot be discussed at a great meeting of representatives, when the object is to secure the recognition of a great principle like institutional treatment. To that principle, and the consequences of its non-inclusion in the National Insurance Bill, speakers will, we hope, confine their remarks, leaving all details for future consideration. They will have to be threshed out, after the passing of the Bill, with the Insurance Commissioners, the Health Committees, and the managers of each voluntary hospital. We congratulate Mr. A. W. West, the chairman, and Dr. D. J. Mackintosh, the honorary secretary, upon the energy and success with which they have worked to make effective the organisation and unification of the voluntary hospital managers throughout the United Kingdom.

### RESIGNATION OF THE CITY MEDICAL OFFICER.

Dr. William Collingridge, Medical Officer of Health for the City, has asked the Corporation to allow him to retire as soon as possible, on the ground that he is physically unfit to carry on the work. Previous to his appointment, which was made in 1901, Dr. Collingridge held the post of Medical Officer to the Port of London. Dr. Collingridge, who is an M.D. of Cambridge, studied at St. Bartholomew's Hospital, has spent much of his spare time with the Volunteers, and served as volunteer surgeon in the Servian Army during the Turko-Servian war in 1875. He has published books on scurvy and plague prevention, besides the sanitary reports of the Port of London. He has also written on the subject of "Quarantine in England."

### THE INSURANCE COMMISSIONERS.

Mr. Lloyd George has been very prompt in announcing the names of those who will be entrusted with the rather thankless task of floating the Insurance Bill in their capacities as Commissioners. The Chairman of the Commission is to be Sir Robert Morant, who leaves the Education Office, where he has been Permanent Secretary for several years, to preside over what may be looked upon as one of the most important State departments of the future. Mr. John Bradbury, C.B., who is principal clerk to the Financial Department of the Treasury, and Mr. D. J. Shackleton, who is at present Labour Adviser to the Home Office, are the other two Commissioners who have had considerable departmental experience, and their appointment will give general satisfaction. Mr. Lister Stead, secretary to one of the largest friendly societies in the kingdom, will suitably represent the large interest which these societies have under the new scheme. In addition the Chief Registrar of Friendly Societies is to be *ex-officio* a member of the Commission. Miss Mona Wilson, who is at present one of the members of the Trade Board appointed under the Trade Boards Act, will watch the interests of the women, but it is somewhat surprising that only one lady Commissioner has been appointed. Mr. J. Braithwaite, assistant-secretary to the Inland Revenue Department, is to be secretary to the Commission. There yet remains the medical Commissioner, whose name is to be announced later. At the present juncture it is idle to speculate as to the choice which the Chancellor may make in filling this important appointment, but the composition of the Commission, as now announced, is on the whole so fair that it may be forecasted that the medical Commissioner will be a gentleman who is thoroughly acceptable to the profession, and who has had some experience of compulsory insurance work. One or two names —notably that of Sir Thomas Oliver—stand out pre-eminently from the number of medical men who are qualified to undertake the duties, but Mr. Lloyd George has probably made up his mind already, and the profession can only adopt the policy of his chief and "wait and see."

### THE NEW MEDICAL OFFICER FOR HACKNEY.

Dr. William Brander, M.D., of Aberdeen University, has been appointed Medical Superintendent of the Hackney Union, which is one of the largest in London. There were some eighty applications for the post, which has fallen to a Northern medical officer, since Dr. Brander has held the post of Superintendent to the Sheffield Workhouse hospital since 1907. Previously to this Dr. Brander has held appointments at Ecclesall and at Middlesbrough. More than one Poor Law appointment has fallen recently to Sheffield men, and the local papers are particularly warm in their congratulations in consequence.

### THE HOSPITAL ALMONERS' COUNCIL.

The inaugural meeting of the newly-constituted Hospital Almoners' Council was held at Caxton Hall on November 24, the Bishop of Stepney, Vice-President, in the chair. The Council, we understand, has considerably increased its membership, and now includes sixteen representatives of the governing bodies and medical staffs of twelve London hospitals. The heads of women's colleges in Oxford, London and Dublin have also joined the Council, together with hospital almoners working in London and others interested in the movement. The Council will continue to select and train women for almoners' posts, and to recommend them to hospitals. At the meeting a constitution was adopted, and an executive committee chosen. The speakers were Mr. C. S. Loch, the founder of the movement, Sir Edward Brabrook (Vice-President), Dr. R. Hutchinson, Dr. T. W. Eden, and Dr. A. Chas. E. Gray, former chairman of the Council. The scope of the hospital almoners' work is likely to extend so much, that the better organisation of this new profession is one which all hospital officers will welcome.

### NO APPLICANTS!

It is not often that an up-to-date hospital finds itself without a single applicant for the two posts of house surgeon and his assistant, yet such has recently been the experience of the Doncaster Infirmary. It must not be hastily concluded that this reflects upon the Institution. It reflects only upon a somewhat tedious process of election which takes four weeks to add to the medical staff. Several applications were received for the post vacated by Dr. R. Anderson and for that of assistant, which the increase of work had necessitated, but during the four weeks before the election was completed these applications were withdrawn. Each candidate presumably had made several applications which received a speedier result than the Doncaster Infirmary methods permitted. Mr. W. E. Lister, the Hon. Secretary, has suggested an alteration in the rules by which the appointment should be made and the salary fixed by the committee, while the medical staff should report on the testimonials. This, he thinks, will expedite matters. Another change is the raising of the salaries by £20 each to £100 and £80 respectively. We shall expect to hear that the old number of applications, which was about sixty, has been restored for posts at Doncaster.

### THE DEADLOCK AT CHESTER.

The rapid transformation of Chester Infirmary, which involves an expenditure of £25,000, of which £17,000 has been subscribed, is now threatened by the City Council's scheme of building a refuse destructor in the immediate neighbourhood of the Infirmary. On the one hand, the Medical Officers of Health, Dr. Currie and Dr. Rennet, allege that the site of the electricity works, if used for the destructor, would not injure the Infirmary, while on the other, the medical committee of inquiry consider that it "would imperil the continued retention of the Infirmary on its present site." A long letter embodying the expert opinions which the Infirmary has been able to collect on its behalf was recently sent to the Council, and one of the most important arguments which it contained referred to the stipulation on which the fulfilment of Mr. Albert Wood's promise of £12,500 depends: that the Infirmary managers be satisfied with the site chosen by the City Council. Mr. J. R. Thompson, the Chairman of the Infirmary, declared that the building of the destructor on the proposed site would mean "the abandonment of the Infirmary extension scheme." Any authority who has visited Chester Infirmary would feel that that contingency should not on any account be allowed to occur.

### NEW LONDON HOSPITAL CHAIRMEN.

Among important new institutional appointments is that of Captain the Hon. Edward Stanley Dawson, R.N., to be Chairman of University College Hospital, in succession to the late Lord Cathcart. Another new chairman is Mr. Arthur Hood, who has been appointed to succeed the late Mr. John Hampton Hale at the head of the Royal Dental Hospital Board, on which he has sat for many years. Another appointment is that of Sir Henry Lopes, Bart., to the position of Treasurer of the Royal Hospital for Incurables, Putney Heath, in succession to the late Mr. H. J. Allcroft. Sir Henry has been a member of the Board for some years, and his acceptance of the treasurership is only the latest sign of his interest.

### GUY'S HOSPITAL "SCANDALS" AND THE SOUTHWARK GUARDIANS.

For six years at least, that is from September, 1905, until the present day, the Southwark Guardians have deliberately refrained from doing their duty to the sick poor of Southwark. A reference to The Hospital of October 7th, 1905, page 3, will show that this statement cannot be questioned. In September 1905 a correspondence took place between the Guardians of Southwark Union and the authorities of Guy's Hospital, which dealt with and disposed of the especial allegations of "scandals and neglect" made against Guy's Hospital at that date. Mr. Cosmo Bonsor, the treasurer, in a letter dated September 28th, 1905, very properly not only rebutted the implication of a scandal at Guy's by a recital of the facts, but expressed on behalf of the Governors and large ratepayers their regret that the Southwark Guardians did not provide infirmary accommodation within the limits of their Union. This is the crux of the whole matter and, as we pointed out in The Hospital of the 5th ultimo, the Southwark Guardians then failed, and have for six consecutive years failed, to do their duty as Guardians of the poor, by making adequate provision for accident and other cases of emergency which they are charged with the duty of succouring. We expressed the hope on the 5th ult. that the Local Government Board would hold a public enquiry into this matter without delay, that the Southwark Guardians might be brought to a sense of this grave neglect of their duties and responsibilities in not having erected many years ago a receiving house for the reception of these cases, equipped with a number of beds and proper medical and nursing provision. Instead of doing this, in 1905 the Guardians tried to compel this work of theirs to be undertaken by the voluntary hospitals, which have neither the means nor is it their province or duty to do such work for Boards of Guardians. Failing in this attempt they tried to bring pressure upon Guy's Hospital by exciting prejudice in the public mind against that institution in the most scandalous way.

### A CRUSADE OF MISREPRESENTATION.

During the last few months an epidemic of similar misrepresentation has filled certain newspapers with paragraphs headed "Another Scandal at Guy's Hospital." How far has this defaulting public authority inspired this crusade? The actual position to-day is soberly and plainly set forth in a letter from Sir Cooper Perry which we publish in another column. In face of the facts that the Southwark Guardians have been clearly in default all these six years past, and that most of the fault of the scandals in question does not rest at the door of Guy's Hospital but at the door of the Southwark Board of Guardians, we venture to appeal to Mr. John Burns, who practically represents the south of the Thames in Parliament, and has so a direct interest in the matter. As President of the Local Government Board he has power to call upon the Southwark Guardians to perform their duty to the sick poor, and to provide them with the effective medical attendance and accommodation, to the absence of which the scandals in question are directly traceable. In any case our contemporaries in the Press, which have dealt with these matters in the past, will, we venture to hope, take care in the future to put the saddle on the right horse. Then Guy's Hospital will be left, as it ought to be left, to discharge its proper duties to the sick, with that efficiency and skill which have deservedly earned for it so great a reputation throughout the Empire. The work of the voluntary hospitals, of which Guy's is one, is far too heavy and important to be subject longer to these scandalous attacks on the part of those who, when judged by their conduct during all these years, cannot justly claim to be the friends and succourers of the sick and suffering south of the Thames.

### THE LATEST THING IN THEATRE UNITS.

We have received from Mr. Charles Postgate, the Secretary-Superintendent of the North Riding Infirmary at Middlesbrough, an extremely interesting pamphlet descriptive of the new theatre block which was opened by Mr. Francis Samuelson, J.P., President of the Infirmary Committee, on Thursday last week. The block, which has been built from the plans of Messrs. R. Lofthouse and Sons, is placed at the extreme south-west of the Infirmary, connected to it by a continuation of the children's ward corridor. The door into the theatre opens from an ante-room, on the right of which is an anæsthetic room, with an *x*-ray room beyond it, while correspondingly on the left are a doctor's room and a sterilising room. These are all described in detail, and excellently illustrated in the pamphlet, which, in noting that the *x*-ray room is entered from the anæsthetic room (an unusual arrangement), remarks that "no inconvenience arises from this fact, as work in the two rooms is carried on simultaneously." The relation of the anæsthetic room to the remainder of the unit obviates any risk of a patient who is about to be operated upon crossing the path of one who, after operation, is being sent back to the ward. This is also a time-saving device when the number of patients to be operated upon is large. Mr. Charles Postgate, who is responsible for the preparation of this descriptive pamphlet, is certainly to be congratulated upon the care and thoroughness with which it has been carried out. We are strongly in favour of such educative methods of consolidating local support, and the cost of the new unit, £2,800, to which Mr. Samuelson subscribed £500, becomes intelligible and interesting to the public, when they are admitted so fully and so pleasantly into the details of the undertaking. Architects, moreover, intent on new appliances, and specialising contractors, will find all the information they require in this account, which is commendably technical and readable throughout.

### THE LEAGUE OF MERCY.

The annual meeting of the Presidents of the League of Mercy will be held at St. James' Palace on Friday, December 15, at 3 o'clock, when Lord Farquhar will preside. Presidents will enter by Ambassadors' Court, and the meeting will have an added interest from the fact that H.R.H. the Duchess of Albany will make a presentation of the Order of Mercy.

### THE REBUILDING OF CHELSEA HOSPITAL.

The site of one and a quarter acres which Lord Cadogan, as announced in The Hospital, recently, gave to the Chelsea Hospital for Women is to be used for the rebuilding of the institution. In addition to this the hospital authorities have received a promise of £10,000 for the same object from the trustees of a charitable bequest. With this send-off it is hoped that the necessary money will be raised. We indicated, at the time Lord Cadogan's gift was announced, a few of the difficulties with which the work in the present building is confronted. We hope that the charitable public will make some effort to help them before it decides to give its money elsewhere. Mr. Herbert Jennings, the secretary, and Mr. Henry Wright, the treasurer, will be glad to supply all information and to receive any subscriptions that the public may send.

### HOSPITAL PROBLEMS AT ARUNDEL.

The Arundel Emergency Hospital is losing the services of its honorary Secretary, Mr. J. Durston, who has held the post for six years, owing to his removal to Bognor, consequent on his promotion. His resignation comes at a time when there is increased demand for the services of the hospital, owing to the growing number of patients received and of operations performed. In this connection some interesting remarks were made last week by Dr. Eustace, who stated that " the institution was not intended, when it was started, to admit everybody who might be taken ill. They had very strictly kept out what might be called chronic cases. Only those requiring drastic treatment were admitted. If it were the desire that the hospital should be used as it had been in the past year, one thing was clear: they must have increased support." It was then agreed, on Dr. Eustace's proposal, that the fee charged for private patients should be raised from one to two guineas. The Duke of Norfolk, who presided at the annual meeting, said that he hoped the formation of the Arundel Hospital would not injure such an old-established institution as the neighbouring hospital at Chichester.

### DR. THOMAS LANDON'S DEATH.

News has just come to hand of the sudden death at Turfontein, Johannesburg, of Dr. Thomas H. W. Landon at the early age of forty-two. All Guy's men remember Dr. Landon as the medical student who founded the Christmas entertainment which is now held every year for the benefit of the children in the district, whose dreary lives made a deep impression on his student days. In spite of his absence in South Africa, Dr. Landon always remembered these children at Christmas, and now six hundred are chosen from the poorest homes for the tea, the entertainment, and the presents of food, clothes, and toys which are distributed to them. The funds for this purpose are collected by Guy's students and others, who thus keep Dr. Landon's memory green every year in Guy's district.

### THE TURF AND A LIVERPOOL HOSPITAL.

The State visit of Lord and Lady Derby as Lord Mayor and Lady Mayoress to the Royal Southern Hospital last Sunday was attended by one amusing incident. On entering the children's ward, which was decorated with his lordship's racing colours, and observing a diminutive jockey astride the children's rocking-horse, Lord Derby immediately promised to give the first stakes won on the turf by any horse of his next year. He is also going to send toys for the children's ward at Christmas. We wonder who the turf enthusiast was who suggested this happy ward decoration, and congratulate Mr. John Rankin, the president, and Mr. Allan Naldrett, the superintendent and secretary, on the enterprise of some member of their staff.

### THE BROWNHILL CASE AT BIRMINGHAM.

A good deal of attention has been attracted to allegations made against the medical staff of the Birmingham General Hospital, owing to their temporary refusal to treat Mrs. Brownhill, whose fingers were injured in a brass founder's factory. On a recent Thursday, it is stated, she met with her accident, was taken to the General Hospital, and according to her own statement kept waiting for a considerable time. On the following Saturday she revisited the hospital, her husband complained of the previous delay, and another doctor dressed her hand. On Monday she saw the latter again, and states that she was told not to come again, and that he would not sign her incapacity for work certificate, which was essential to her claim for compensation. On Wednesday the same refusals are alleged. In the meantime, Mrs. Brownhill's case had been taken up by Canon Mackenzie, who received from Mr. Howard Collins, house governor, a letter, saying that the delay complained of was no more than justice to previously waiting patients required, and that the refusals to treat her hand and to sign her certificate were due respectively to her rudeness to the doctor, and to the hospital's practice in filling up only those forms which itself provides. Mrs. Brownhill found another champion, however, in the *Birmingham Evening Dispatch*, which obtained an undertaking from Mr. Collins that if an apology was offered he would ask the doctors to reconsider their decision. The story is that they refused to do so. Last Friday Mrs. Brownhill was promised treatment by the casualty officer. We cannot believe, however, that an apology, if promptly offered, was not accepted by the authorities, for they must have been fully alive to the fact that to continue to refuse treatment to a woman who, because of her injury, and therefore in spite of any faults, might command so easily the hasty support of the public.

### THE OUT-PATIENT QUESTION AT LEEDS.

We understand that the Leeds General Infirmary is endeavouring now to grapple with the perennial if usually neglected problem of its out-patient department. That department, like every other one at which an enormous number of cases have to be examined, has entailed an unenviable wait, a long delay on the out-patients which can ill be afforded by the working-classes which form the great majority. To remedy this in some degree has been the worthy object of consideration, and the proposal most likely to achieve this is one for greater specialisation. In other words an additional honorary surgeon is suggested, while the ophthalmic and aural sub-departments will be separated and placed under the control of two honorary surgeons. The suggestion is not a new one, but the problem it was offered to solve does not get less pressing, and instead of coming into force next March at the General Board meeting, a special meeting has been called to consider the advisability of bringing the proposals into force immediately. The Leeds Infirmary has deserved praise on many occasions, and this proposal is one which is to be commended, for the lot of out-patients in certain respects has been an exceedingly trying one which no general steps have been taken to amend. As long as we have out-patient departments something must be done to obviate their general discomforts and delays.

### THE RUGBY HOSPITAL EXTENSION.

The need of a new nurses' home at Rugby Hospital will probably be satisfied in the new year, since the purchase of a house and five and a half acres of land adjoining the hospital has been decided on. The purchase and equipment is expected to cost £4,500, of which £1,000 has been promised by Mr. Arthur James, and other subscriptions to about half the required sum are forthcoming. It will be seen that the scheme has importance not so much from the house as from the land which it includes. This can be made valuable to the hospital in a variety of ways, and we shall look forward to hearing what purpose it will be made to serve.

### OPTICIANS AND EYE SPECIALISTS.

The Institute of Ophthalmic Opticians is agitating itself over a proposal whether or not to form classes of instruction in ophthalmology for the benefit of its members, not one in a hundred of whom, we are assured by Mr. F. W. Bateman, has the slightest knowledge of diseases of the eye. The question surely can be decided by an inquiry as to whether this want of knowledge has hindered in any way the power of opticians to carry out the prescriptions of ophthalmic surgeons who send formulas to them to be made up. No doubt differing opinions on this point will be held, but there is little doubt that a knowledge of ophthalmology would lead opticians to prescribe as well as to construct various forms of glasses, in precisely the same way as a knowledge of the properties of drugs has led chemists and druggists to become the poor man's doctor and to prescribe for him, or to sell to him those patent medicines which have the medical advice wrapped round the bottle. No assurances will convince us that the human nature of opticians is any less ready to act on its knowledge than that of chemists has proved to be; and any attempted boycott of the prescribing optician will fail as surely as it would do if tried on the chemist and druggist, as some practitioners have been known to suggest.

### THE INFIRMARY BIRTHDAY AT DERBY.

Peculiar interest was excited at Derby last week in connection with the celebration of the Infirmary's 102nd anniversary. The annual meeting, which was held on the same day, was presided over by the Duke of Devonshire, who, after referring to the complicated nature of the Insurance Bill, said that a strenuous effort must be made to preserve the voluntary hospitals. Mr. H. Strutt, the chairman of the weekly board, who followed, deprecated the debts on the building and expenditure accounts, and said that an additional income of £2,000 was called for. The remaining speakers included Mr. G. H. Smith, treasurer of the Convalescent Home; Mr. G. Brigden, the Mayor; Mr. W. H. Richardson; Mr. M. Attwood, treasurer; Mr. G. Innes, Mr. A. Cox, Colonel Buchanan, and Mr. J. Cartwright and Mr. J. P. Hudson, working-men representatives. After a piece of ground had been voted as a gift for the site of a Florence Nightingale memorial, the wards were visited, in which a Victoria incubator, the money for which has been collected and personally given by the wife of the gynæcologist, Mr. Hicks, created an amount of interest. The Infirmary authorities may be congratulated on the local interest that was shown on this occasion, which changed a semi-formal gathering into an interesting and varied one.

### THE CHARITIES BILL IN AUSTRALIA.

The problem how best to regulate the collection and distribution of the large sums of money which are given every year to charitable institutions is solved in this country by free competition, tempered, it is true, by the inquisition of one or more voluntary agencies, and, in the case of money bequeathed, supervised in some degree by the Charity Commissioners. In Australia a Charities Bill is being discussed. Its main proposal is the appointment of a board of three, who, it seems, would have certain powers over the management of existing institutions, besides rights of closing and amalgamation, together with the allocation of all the

Government or private money that it might receive. It is an heroic attempt to grapple with a difficult problem. One can imagine that the duties of the dramatic censor would be as nothing to the examination of proposed charitable agencies which the new commissioners would have to go through. Imagine the difficulty and responsibility of saying that a new kind of special hospital were not needed—and the weariness of examining every new philanthropic enterprise propounded.

### A SURPRISE VISIT TO BENENDEN.

We understand that her Royal Highness Princess Christian paid a surprise visit to Benenden Sanatorium last Saturday afternoon, and visited the wards, administration buildings, dining and recreation halls. Her Royal Highness then inspected the gardens where the patients are under treatment by a system of graduated work, which trains them during their convalescence to a condition of work-fitness and enables the successful cases to resume their wage-earning occupation without much fear of a relapse. Patients were employed at gardening and carpentry in the open-air workshops, while those completing their treatment were seen performing heavy tasks. In the earlier stages of treatment such light tasks as wood-chopping and weeding are prescribed. Princess Christian spoke to each bed patient, and in leaving expressed her keen interest in the work. Her Royal Highness was received by the chairman of the Institution, Mr. C. H. Garland, and the hon. advisory physician, Dr. T. D. Lister, who were themselves present on a visit of inspection. The medical superintendent, Dr. J. D. Macfie, Dr. G. M. Mayberry, assistant medical superintendent, and the Secretary, Mr. Henry Seagrave, were also presented. There is no doubt that such sanatoria as Frimley and Benenden, where the graduated work system prevails, must systematically have achieved very satisfactory results, and these not only with incipient cases.

### THE HEALTH OF THE WARDMAID.

In the majority of households, at any rate among the middle class, the domestic servant is a young girl, drawn from the upper working class—a class generally ignorant of simple rules of health, of the need for regular hours of sleep, of regular diet, and of that attention to minor ailments which may prevent more serious disease developing. Add to this the carelessness of self which is so characteristic of youth throughout the animal kingdom, and we see that the domestic servant is one who needs some guidance. The discussion on the Insurance Bill has brought out the fact that in the large proportion of cases the employer sees to the attendance of a doctor when a domestic servant is ill, and there can be no doubt that most mistresses are kindness itself when actual illness attacks the maidservant. But we think more might be done by the mistresses to help the girl in time of health than is done at present. Frequently the mistress is to blame in putting conditions on the servant which are actually deleterious to health. We would refer particularly to the question of sleep. A young woman between the age of seventeen and twenty-five needs eight hours of sleep every night. This is necessary even in the healthy life of the middle-class girl with her walk, her bicycle, and her game of hockey; much more is it so in the case of the girl under discussion who works hard all day, frequently in the hot atmosphere of a kitchen or in the microbe-laden cloud of dust of a swept carpet, and who goes out twice a week. If an hour is allowed in the bedroom for dressing, undressing, and ablutions, the time during which the girl should be in her bedroom is nine hours every night.

### LONDON DISPENSARIES AND A SANATORIUM FUND.

The growth of dispensaries for the prevention of consumption, especially in London, is leading inevitably to a demand for sanatoria where the preliminary treatment given by the dispensaries may be prolonged, and also that institutional treatment may be found immediately for suitable cases. Much work of this sort has been carried on by the C.O.S., which now finds itself in some financial difficulty and is appealing for funds in order that the work may be continued. The success of this work is shown by the fact that of all the patients sent away since 1902 for treatment, 53 per cent. are now working or are fit to work. The dispensaries inevitably rely, and will rely increasingly—for the Government insurance proposals are still not understood, and must, to say the least, mature slowly—on such help as the C.O.S. has given. A cessation of its work, therefore, just now would be a double loss to anti-phthisis effort. Donations marked "Sanatorium Fund" should be sent to the Secretary at 296 Vauxhall Bridge Road, S.W.

### THIS WEEK'S DRUG MARKET.

Quiet conditions continue to prevail in the drug market, and few changes in value have taken place during the week. The demand for quinine has not been so brisk, but a fair quantity has been sold, and it is not improbable that makers will advance their prices. It is, in fact, rumoured that there is a possibility of an arrangement between growers of cinchona bark and makers of quinine the effect of which would be to restrict the output and, consequently, raise the price of the alkaloid; similar rumours have been current on previous occasions, so that too much importance must not be attached to the present report, although the fact remains that the price tendency is in an upward direction. Opium maintains its strong position, as also does morphine; makers of codeine have advanced their prices, as was not altogether unexpected in view of the high cost of the raw material. Citrates, including iron and ammonium citrate and iron and quinine citrate, are slightly dearer. Sugar of milk is gradually receding in price. Hydrastis canadensis is now in slightly better supply, and as a consequence is lower in price. Balsam of tolu is again rather dearer. Oil of orange is dearer.

